# Angiogenic potential of endothelial progenitor cells and embryonic stem cells

**DOI:** 10.1186/2045-824X-3-11

**Published:** 2011-05-11

**Authors:** Peter C Rae, Richard DW Kelly, Stuart Egginton, Justin C St John

**Affiliations:** 1 Centre for Cardiovascular Sciences, College of Medical & Dental Sciences, University of Birmingham, UK; 2 Clinical Sciences Research Institute, Warwick Medical School, University of Warwick, UK; 3 Centre for Reproduction & Development, Monash Institute of Medical Research, Clayton VIC 3168, Australia

## Abstract

**Background:**

Endothelial progenitor cells (EPCs) are implicated in a range of pathological conditions, suggesting a natural therapeutic role for EPCs in angiogenesis. However, current angiogenic therapies involving EPC transplantation are inefficient due to rejection of donor EPCs. One solution is to derive an expanded population of EPCs from stem cells *in vitro*, to be re-introduced as a therapeutic transplant. To demonstrate the therapeutic potential of EPCs we performed *in vitro *transplantation of EPCs into endothelial cell (EC) tubules using a gel-based tubule formation assay. We also described the production of highly angiogenic EPC-comparable cells from pluripotent embryonic stem cells (ESCs) by direct differentiation using EC-conditioned medium (ECCM).

**Results:**

The effect on tubule complexity and longevity varied with transplantation quantity: significant effects were observed when tubules were transplanted with a quantity of EPCs equivalent to 50% of the number of ECs originally seeded on to the assay gel but not with 10% EPC transplantation. Gene expression of the endothelial markers VEGFR2, VE-cadherin and CD31, determined by qPCR, also changed dynamically during transplantation. ECCM-treated ESC-derived progenitor cells exhibited angiogenic potential, demonstrated by *in vitro *tubule formation, and endothelial-specific gene expression equivalent to natural EPCs.

**Conclusions:**

We concluded the effect of EPCs is cumulative and beneficial, relying on upregulation of the angiogenic activity of transplanted cells combined with an increase in proliferative cell number to produce significant effects upon transplantation. Furthermore, EPCs derived from ESCs may be developed for use as a rapidly-expandable alternative for angiogenic transplantation therapy.

## Background

In the early embryo, mesodermal stem cells in the bone marrow (BM) differentiate to form haemangioblasts, the common precursor of haematopoietic stem cells and endothelial-lineage angioblasts [1,2]. During vasculogenesis these immature but lineage-committed angioblasts, termed endothelial progenitor cells (EPCs), migrate and congregate into clusters, called blood islands, forming the primary vascular plexus from which a complex microcirculation arises [3,4]. In contrast, adult vascular growth occurs primarily through angiogenesis whereby new capillaries develop endogenously from fully-differentiated endothelial cells (ECs) within existing vessels [5]. However, angiogenesis is not the sole mechanism by which the adult vasculature is augmented [6,7]. Circulating EPCs share phenotypic characteristics with embryonic EPCs [8] and incorporate into sites of neovascularisation, suggesting a role for EPCs in angiogenic renewal [9,10]. They express endothelial-specific markers, including vascular endothelial growth factor receptor 2 (VEGFR2), CD31, CD133, VE-cadherin and von Willebrand factor (vWF), which have various roles in cell-cell adhesion, vascular permeability and the modulation of other cellular responses during angiogenesis [11,12]. Indeed, EPCs are implicated in angiogenesis stimulated by conditions such as coronary artery disease and myocardial infarction, confirmed by clinical observations of EPC mobilisation in such patients and incorporation into foci of pathological neovascularisation [13,14].

Nevertheless, current approaches to angiogenic therapies are problematic. Endogenous approaches most likely rely on the recruitment of circulating EPCs, and the delivery of a single pro-angiogenic substance is insufficient to elicit the complete and prolonged response necessary for effective angiogenesis [15,16]. Exogenous therapies involve administration of allogeneic donor EPCs, which with poor HLA matching leads to increased immune rejection resulting in reduced transplantation efficiency [17,18]. Consequently, the use of an expanded population of autologous EPCs from the patient's own adult stem cell population is desirable.

In contrast to EPCs, the inner cell mass of blastocyst-stage embryos gives rise to a population of self renewing pluripotent, embryonic stem cells (ESCs) [19], which are precursors to all cell types of the body [20]. Whilst potential ethical considerations must be considered, such as the use of otherwise viable human embryos for cell harvesting, the capacity of ESCs for unlimited growth may allow rapid *in vitro *expansion and differentiation, producing endothelial-like cells for transplantation. Differentiation can be induced either spontaneously or directed specifically towards the endothelial lineage using EC-conditioned medium (ECCM). This medium would require specific growth factors, such as VEGF, basic fibroblast growth factor (bFGF), epidermal growth factor (EGF), interleukin-6 and erythropoietin to promote differentiation [21-23].

Chemical stimuli may, at least in part, drive the response of EPCs during angiogenesis. These stimuli can be released from surrounding tissues, i.e. from within the microenvironment, or from the endothelial cells themselves. Indeed, it has been shown in hypoxic wounds of diabetic patients that EPCs in the BM respond by following chemokine gradients, resulting in their homing to sites of hypoxia where they can participate in neovascularisation [24]. Consequently, the microenvironment in which progenitor cells are cultured is critical to their ability to maintain their progenitor status, i.e. to self-renew and give rise to differentiated cell types as and when recruited to do so. For instance, it has been shown that under the appropriate microenvironmental cues, achieved by using a combination of *in vitro *culture supplements, cord blood-derived precursor cells are able to give rise to cells characteristic of EPCs [25]. Furthermore, specific Notch signals within the BM microenvironment have been demonstrated to be vital for EPC-mediated vasculogenesis through murine knockout models of hindlimb ischaemia [26].

Although the remodelling of mature ECs during the development of endothelial tubules *in vitro *is well understood, the mechanism of EPC involvement remains unclear. *In vitro *assays are able to model the *in vivo *microenvironment, and those cues specific to angiogenesis enable us to understand how related cell types form tubules and networks of branches. They also allow us to ascertain the concomitant expression of lineage-specific genes during development and tissue repair. For example, it has been shown that ECs form organised tubule networks when cultured on glycoprotein-rich matrices containing angiogenesis-stimulating growth factors [27], and their potential to form pseudo-vessels can be demonstrated using an *in vitro *gel-based assay [28]. Here, we have determined, in a time-dependent manner, how EPCs form tubule networks *in vitro *using an ECMatrix gel. We then showed how ESCs are able to mimic this outcome when cultured in a defined medium to promote EPC differentiation. We also demonstrated that EPCs transplanted into gel-based cultures of ECs that have begun to exhibit tubule loss and reduced network density can rescue tubule formation, by generating new tubules rather than incorporating into existing tubules.

## Methods

### Cell lines and culture media

The murine EC line MCEC-1 was provided by Prof. Gerard Nash (University of Birmingham, UK). The murine EPC line MFLM-4 was obtained from Seven Hills Bioreagents (Ohio, US). Endothelial growth medium consisted of Dulbecco's modified Eagle medium (DMEM) with 10% fetal bovine serum (FBS), 1% penicillin-streptomycin and 2 mM L-glutamine, supplemented with 10 U·ml^-1 ^heparin and 0.1 μg·ml^-1 ^recombinant murine epidermal growth factor (EGF) (for MCEC-1) or 25 mg·ml^-1 ^amphotericin B and 10 ng·ml^-1 ^basic fibroblast growth factor (bFGF) (for MFLM-4). ESCs derived from blastocysts of 129S2/SvPas mice (D3) were obtained from American Type Culture Collection (ATCC, Middlesex, UK). ESC growth medium consisted of Knockout DMEM (Invitrogen, Paisley, UK) containing 15% ESC-Screened FBS (HyClone; Thermo Fisher, Leicestershire, UK), 1% penicillin-streptomycin, 2 mM L-glutamine, 1% non-essential amino acids, 0.1 mM β-mercaptoethanol and 10 ng·μl^-1 ^recombinant murine leukemia inhibitory factor (LIF) (Chemicon, Livingston, UK). Undifferentiated ESCs were maintained on CF-1 murine embryonic fibroblast feeder cells (ATCC) mitotically-inactivated by treatment with 10 μg·ml^-1 ^mitomycin C for 2 h at 37°C.

### Differentiation of stem cells

ESCs were spontaneously differentiated using the hanging droplet method [29]. Undifferentiated cells (D0) were resuspended in growth medium (minus LIF) and plated as 20 μl droplets (450 cells/drop) on Petri dishes. Inverted dishes were incubated at 37°C/5% CO_2 _for 48 hours (D1-2) to induce embryoid body (EB) formation. EBs were then grown in suspension for 5 days (D3-7). For directed differentiation, cells were resuspended in ECCM for D3-7; ECCM was produced by 24 h incubation with MCEC-1 cells.

#### Lectin staining and uptake of acetylated (ac-)LDL

EPCs and ECs were incubated with 0.5 mg·ml^-1 ^fluorescein-conjugated *Griffonia (Bandeiraea) simplicifolia *lectin I (Vector Laboratories Inc., California, USA) in PBS for 1 h at 37°C, and 10 μg·ml-1 of 1,1'-dioctadecyl-3,3,3',3'-tetramethylindocarbocyanine perchlorate (DiI)-labelled ac-LDL in culture medium for 4 h at 37°C.

### *In vitro *tubule formation assay

Angiogenic activity of cells was assessed using the *In vitro *Angiogenesis Assay Kit (Chemicon). For each assay, 3 μl Diluent Buffer was added to 27 μl ECMatrix Gel Solution and polymerized on an 18 mm glass coverslip by incubation at 37°C for 30 minutes. Cells were seeded at a density of 8 × 10^4 ^cells/coverslip in 400 μl growth medium and returned to the incubator for 14 h. Images were recorded every 2 h for tubule quantification.

### *In vitro *EPC transplantation

Transplantation was performed by addition of EPCs into a tubule formation assay containing ECs. Prior to transplantation, EPCs were labelled using 5 mM Qtracker 655 quantum dot (Qdot) labelling solution (Invitrogen, Paisley, UK) according to manufacturer's instructions. To investigate the effect of cell number on transplantation two quantities of EPCs were used, equivalent to 10% (8 × 10^3 ^cells) or 50% (4 × 10^4 ^cells) of the number of ECs originally seeded. At 5 h, a suspension of 10% or 50% Qdot-labelled EPCs in 50 μl PBS was pipetted evenly over the EC-containing ECMatrix gel and the coverslip was returned to the incubator.

### Quantification of *in vitro *tubule formation

Tubule formation was quantified by counting nodes, points at which branches intersect, and measuring branch lengths. Nodes were graded based on structure: branch end-points as N1; intersections as N2; junctions of three or four branches as N3 or N4; and nodes of five or more branches as N5+. The average number of each node type was calculated based on five fields of view every 2 h. Branch length was measured using AQuaL Angiogenesis Quantification software [30]. Tubule images were tagged with markers, defined as end-points or junctions, and overall lengths automatically calculated based on five fields of view.

### Gene expression analysis

Cells were recovered from ECMatrix gel using Cell Recovery Solution (BD Biosciences, Oxford, UK). Total RNA was isolated using RNAqueous-4PCR Kit (Ambion, Warrington, UK) followed by decontamination with DNase I (Ambion). Reverse transcription was performed using MMLV reverse transcriptase (Bioline, London, UK) with Oligo (dT)_18 _primers to produce complementary DNA (cDNA). cDNA was used for quantitative real-time PCR (qPCR) performed in a Corbett Rotor-Gene 3000 cycler (QIAGEN, West Sussex, UK) using SYBR Green chemistry. Each qPCR reaction (25 μl final volume) consisted of 1 μl cDNA template, 12.5 μl Quantace SensiMix (Bioline, London, UK), 0.5 μl 50× SYBR Green I Solution (Bioline), 0.2 μM each of forward and reverse primers (Invitrogen) and PCR-grade H_2_O. A 270 bp product of *VEGFR2 *cDNA was amplified with VEGFR2-F (5'- TCTGTGGTTCTGCGTGGAGA-3') and VEGFR2-R (5'-GTATCATTTCCAACCACCCT-3'); a 244 bp product of *CD31 *cDNA was amplified with CD31-F (5'-CGCACCTTGATCTTCCTTTC-3') and CD31-R (5'-AAGGCGAGGAGGGTTAGGTA-3'); a 122 bp product of *VE-cadherin *cDNA was amplified with VE-cadherin-F (5'-TCCTCTGCATCCTCACCATCACA-3') and VE-cadherin-R (5'- GTAAGTGACCAACTGCTCGTGAAT-3'); and a 242 bp product of *β-actin *cDNA was amplified with β-actin-F (5'-CACCACACCTCCTACAATGAGC-3') and β-actin-R (5'- TCGTAGATGGGCACAGTGTGGG-3'). Initial denaturation was performed at 95°C for 15 minutes, followed by 50 cycles of: denaturation at 95°C for 10 seconds; annealing at 55°C (*VEGFR2 *and *CD31*) or 57°C (*VE-cadherin *and *β-actin*) for 15 seconds; and extension at 72°C for 15 seconds. A range of known standards (2 ng·μl^-1 ^to 2 × 10^-9 ^ng·μl^-1^) of each PCR product were generated to enable quantification of gene expression, with the housekeeping gene β-actin used as a calibrator for relative quantification.

### Immunocytochemistry (ICC)

Cells were fixed using 4% paraformaldehyde and non-specific blocking performed using 2% bovine serum albumin, 100 mM glycine and 0.2 mg·ml-1 sodium azide for 30 minutes at room temperature. Primary antibodies included VEGFR2 (goat, 4 μg·ml-1), CD133 (rabbit, 2 μg·ml-1) and CD34 (rat, 5 μg·ml-1) (Abcam, Cambridge, UK). Secondary antibodies included anti-goat conjugated to Alexa Fluor (AF) 488 (rabbit; 1:400), anti-rabbit (AF 594, donkey; 1:500) and anti-rat (AF 594, chicken; 1:400) (Molecular Probes; Invitrogen). Coverslips were mounted using Vectorshield DAPI Mounting Medium (Vector Laboratories Inc., Peterborough, UK). Microscopy was performed using a Zeiss 510 Meta confocal microscope (Carl Zeiss Ltd., Hertfordshire, UK) with ×63 oil DIC objective (NA 1.4) with the detection pinhole adjusted to 1 Airy unit. DAPI was excited at 360 nm and detected at 460 nm, Alexa Fluor (AF) 488 was excited at 488 nm and detected between 515-545 nm, and AF 594 was excited at 594 nm and detected at 617 nm. Qdot-labelled EPCs were visualized by excitation between 405-615 nm and detection at 655 nm.

### Statistical methods

Statistical differences were calculated using one-way ANOVA and post hoc multiple comparisons using the Bonferroni test.

## Results

### Endothelial-specific gene expression is dynamic during lineage development

**Figure 1:**
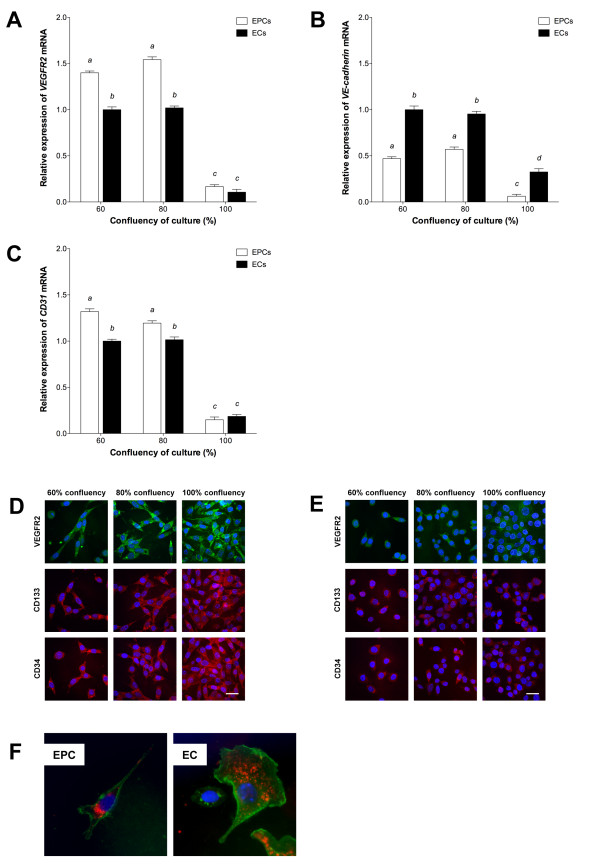
**Characterization of EPCs and ECs**. qPCR of (A) *VEGFR2*, (B) *VE-cadherin *and (C) *CD31 *expression at increasing confluency, relative to 60% confluent ECs (mean ± SEM; n = 3). Columns with different letters are significantly different (P < 0.05). ICC of VEGFR2, CD133 and CD34 in (D) EPCs and (E) ECs. Scale bar = 30 μm. (F) Uptake of DiI-ac-LDL (red) and staining with lectin (green). Nuclear staining (blue) with DAPI-containing mounting medium.

Highly-confluent cells, including the endothelium, demonstrate a reduced response to specific growth factors and proliferative signals such as VEGF [31,32] and contact inhibition is thus implicated as an important endothelial anti-proliferative mechanism. Hence, to determine the effect of contact inhibition on endothelial-specific expression, we analysed the patterns of expression of three key endothelial markers, *VEGFR2, VE-cadherin *and *CD31 *in MFLM-4 EPCs and MCEC-1 ECs cultured at 60%, 80% and 100% confluency. Both cell types expressed all three markers at each stage of confluency, though with significant differences whereby increased confluency, and thus contact inhibition, resulted in significant overall decreases in expression (P < 0.01). To this extent, the relative expression of *VEGFR2 *was significantly greater at 60% and 80% confluency in EPCs than ECs (P < 0.05; Figure 1a). Expression in EPCs increased, though non-significantly, as culture confluency increased from 60% to 80%, whilst no difference in expression was observed between 60% and 80% confluent ECs. Relative expression of *VE-cadherin *was significantly higher in 60% confluent ECs than in the other cells analyzed (P < 0.05; Figure 1b) and decreased with confluency. *VE-cadherin *expression in EPCs increased between 60% and 80% confluency. Expression of *CD31 *was greatest in 60% confluent EPCs but reduced as EPCs became 80% confluent (Figure 1c). *CD31 *expression was also not significantly different between 60% and 80% confluent ECs. Through ICC staining, we were able to confirm expression of VEGFR2, CD133 and CD34 proteins in EPCs (Figure 1d) and ECs (Figure 1e) with little evidence for change with degree of confluency.

Further to the characterisation of the chosen cell lines by gene expression analysis, the endothelial phenotypes of MFLM-4 EPCs and MCEC-1 ECs was demonstrated by endothelial-specific lectin staining and uptake of DiI-labelled ac-LDL (Figure 1f).

### EPCs and ECs demonstrate angiogenic potential by *in vitro *tubule formation

**Figure 2:**
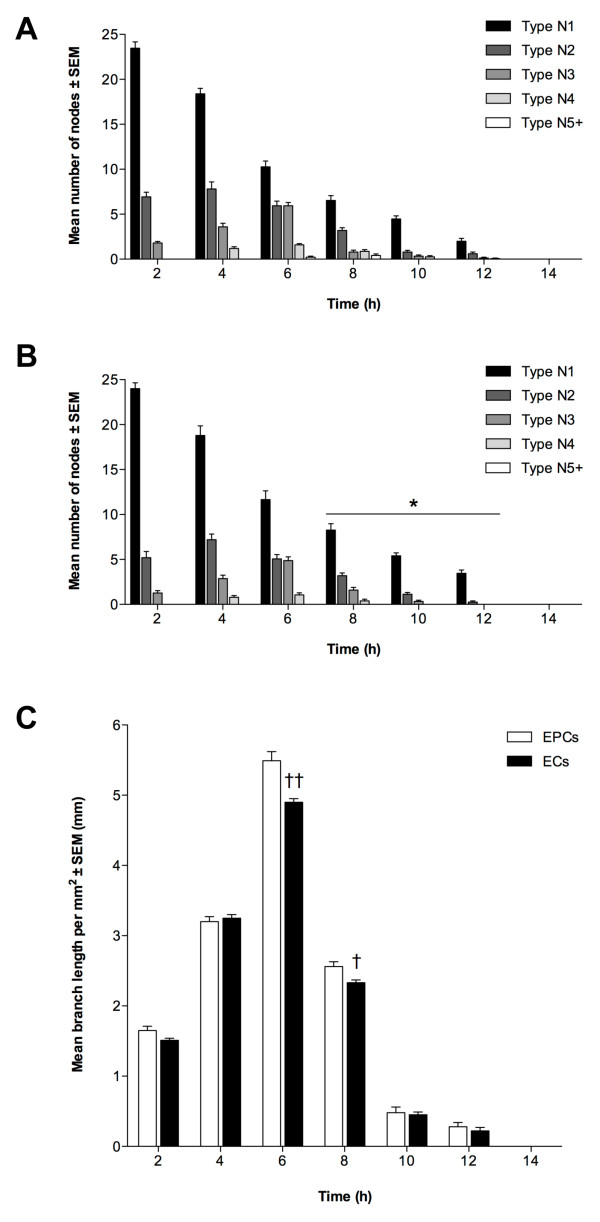
**Angiogenic potential of EPCs and ECs**. Tubule formation of (A) EPCs and (B) ECs quantified by node counting. Presented as mean node number ± SEM (n = 3); *P < 0.05 vs. EPC nodes at same time-point. (C): Branch measurements, as mean length per mm^2 ^± SEM (n = 3); †P < 0.05, ††P < 0.01 vs. EPC branch length at same time-point.

To determine whether EPCs and ECs possessed angiogenic potential, and were thus able to form endothelial tubules, we cultured both cell types individually on ECMatrix gel for 14 h. Tubule formation was quantified by node counting and branch length measurement. The mean number of each node type at each time-point throughout the assay was similar for EPCs and ECs (P > 0.05; Figure 2a and 2b, respectively). A large number of N1 nodes was observed at 2 h, decreasing as the assay progressed, resulting in the formation of more N2 and N3 nodes and thus more complex structures. These were maximal at 4 h and 6 h, respectively for both cell lines. After 6 h, the number of N2 and N3 nodes decreased and they were last evident between 10 h and 12 h. Nevertheless, this gave rise to the formation of more complex structures, N4 nodes, which were first observed at 4 h for both EPCs and ECs and reached a maximum at 6 h and persisted until 8 h (ECs) and 10 h (EPCs) (P < 0.05). In turn, the most complex nodal formation (N5+) was first evident in EPC colonies between 6 to 8 h whilst ECs did not produce these structures. However, no tubule networks were observed after 12 h for either EPCs or ECs. The pattern of tubule formation indicated by branch length measurement was also similar for EPCs and ECs (Figure 2c). Mean length increased for both cell types from 0 to 6 h with maximal branch length being at 6 h, although mean length of EPC tubules at 6 h was significantly greater (P < 0.01). Nevertheless, mean branch length then decreased with significantly lower length being observed for ECs at 8 h (P < 0.05) when compared to EPCs. Thereafter, mean branch length continued to decrease for both cell types.

**Figure 3:**
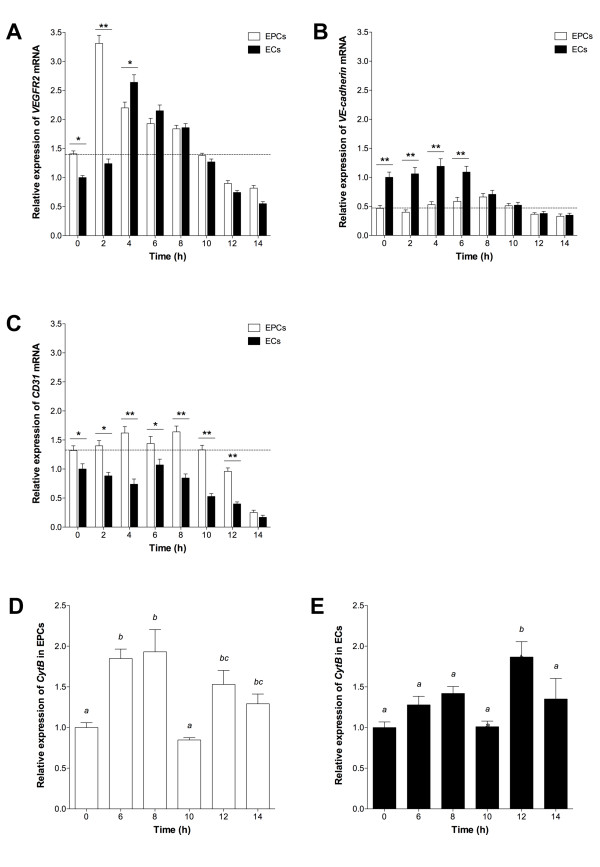
**Gene expression during tubule formation**. (A) *VEGFR2*, (B) *VE-cadherin *and (C) *CD31 *mean expression in assayed EPCs and ECs, relative to 60% confluent ECs (± SEM; n = 3). Dotted line indicates expression in 60% confluent EPCs. Significant differences between cell types indicated (*P < 0.05, **P < 0.01). CytB expression in (D) EPCs and (E) ECs, relative to 0 h. Columns with different letters are significantly different (P < 0.05).

To determine whether tubule formation from EPCs and ECs was a function of endothelial-specific expression, we analyzed *VEGFR2, VE-cadherin *and *CD31 *expression. *VEGFR2 *expression increased in both cells from 0 h to 2 h, with a significantly greater level in EPCs at 0 h (P < 0.05) and 2 h (P < 0.01; Figure 3a) whilst maximal expression was observed at 2 h and 4 h for EPCs and ECs, respectively, followed by a progressive decrease. From 0 h to 6 h, ECs expressed significantly more *VE-cadherin *than EPCs and, following an initial decrease between 0 h and 2 h in EPCs, expression progressively increased up to 8 h (P < 0.05; Figure 3b). Expression in ECs reached its maximum by 4 h (P < 0.05) and then decreased from 4 h onwards whilst in EPCs this started at 8 h, there being no significant difference between EPCs and ECs from this point onwards. Expression of *CD31 *in EPCs increased from 0 h to 4 h (P < 0.05) followed by a reduction at 6 h (P < 0.05; Figure 3c). Conversely, *CD31 *expression in ECs decreased from 0 h to 4 h before increasing to its maximum at 6 h, whilst in EPCs maximal expression was observed at 8 h (P < 0.05). After 8 h, expression in both cell types decreased progressively.

In order to confirm that EPCs were able to develop into fully differentiated cells as they contributed to tubule formation, we analysed the number of mitochondrial (mt)DNA copies over the 14 h assay period. This is indicative of mitochondrial number and their ability to generate sufficient levels of ATP. Although EPCs possessed 30% fewer copies of mtDNA at 0 h, they accumulated similar levels to ECs by 6 h and assumed a similar profile from then onwards (data not shown). A similar outcome was observed for the expression of the mtDNA-encoded gene *cytochrome c oxidase B *(*Cyt B*), although levels were slightly higher when tubule formation was most prevalent (6 h to 8 h; Figure 3d-e).

### Derivation of endothelial-like cells from ESCs can by accelerated using EC-conditioned medium

**Figure 4:**
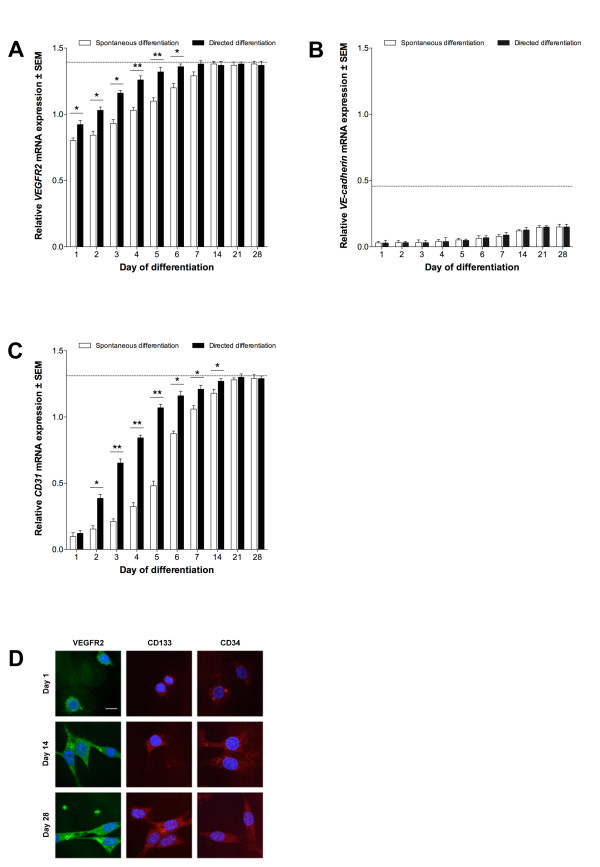
**Endothelial differentiation of stem cells**. (A) *VEGFR2*, (B) *VE-cadherin *and (C) *CD31 *expression in ESCs, relative to 60% confluent ECs (± SEM; n = 3). Dotted line indicates expression in 60% confluent EPCs. Directed differentiation with ECCM, significant differences between treatments indicated (*P < 0.05, **P < 0.01). (D): VEGFR2, CD133 and CD34 proteins in ECCM-treated ESCs. Scale bars = 10 μm.

To determine whether ESCs could give rise to endothelial-like cells, we differentiated D3 ESCs over 28 days using spontaneous and directed methods employing the hanging droplet technique. *VEGFR2 *was detected in ESCs from day (D)1 onwards in both approaches. *VEGFR2 *expression exceeded levels observed in ECs after D4 in spontaneously-differentiated ESCs (P < 0.01) and D2 in directed-differentiated ESCs (P < 0.05; Figure 4a). Expression of *VEGFR2 *in ESCs was significantly different between differentiation treatments from D1 to D6. At D7 and D14, directed- and spontaneously-differentiated ESCs, respectively, expressed levels of *VEGFR2 *similar to EPCs and this continued for the remainder of the culture period. *VE-cadherin *expression was low throughout differentiation of ESCs, with expression not reaching a level comparable to either EPCs or ECs in either treatment before D28 (P > 0.05; Figure 4b). There was no significant difference between differentiation treatments. *CD31 *transcripts were present in spontaneously- and directed-differentiated ESCs from D1 with expression exceeding EC-equivalent levels at D7 (P < 0.05) and D5 (P < 0.01), respectively (Figure 4c). *CD31 *expression in D21 and D28 ESCs (using both treatments) was similar to that in EPCs. Furthermore, *CD31 *expression was significantly different between ESC treatments from D2 to D14 (P < 0.05). ICC confirmed that protein was present for all three genes throughout differentiation for both treatments (Figure 4d).

### Differentiated ESCs demonstrate angiogenic potential comparable to EPCs

**Figure 5:**
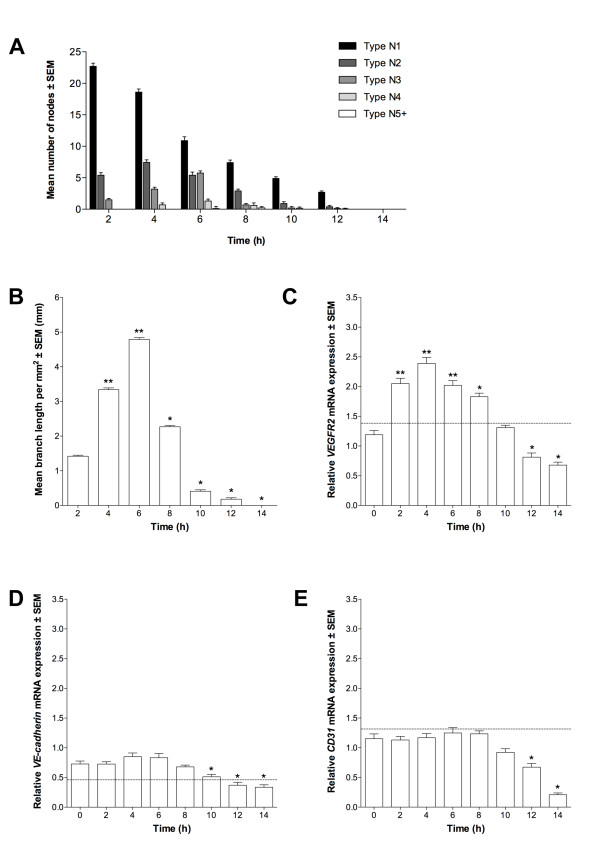
**Characterization of differentiating ESCs at D7**. Tubule formation as (A) node number ± SEM (n = 3) and (B) tubule branch length ± SEM (n = 3; *P < 0.05, **P < 0.01 vs. 2 h). (C) *VEGFR2*, (D) *VE-cadherin *and (E) *CD31 *mean expression in D7 ESCs (n = 3) relative to 60% confluent ECs; *P < 0.05, **P < 0.01 vs. 0 h. Dotted line indicates expression in 60% confluent EPCs.

As directed-differentiated ESCs more rapidly adopted patterns of EPC-associated gene expression, we cultured differentiating D7 ESCs on ECMatrix gel to assess their angiogenic potential. The pattern of nodes formed by D7 ESCs was similar to that of both EPCs and ECs (P < 0.05; Figure 5a). To this extent, the number of N1 nodes was maximal at 2 h and decreased throughout the assay. Nevertheless, the number of N2, N3 and N4 nodes increased to their maximum towards the assay's mid point, whilst N5+ nodes were only evident at 8 h. Branch length measurements of D7 ESCs, as for the node data, were similar to EPCs and ECs (P < 0.05; Figure 5b). Mean length increased from 0 h to 6 h, with maximal length observed at 6 h, and no branches were evident by 14 h.

Assayed D7 ESCs were analyzed by qPCR. Expression of *VEGFR2 *in D7 ESCs increased significantly between 0 h and 2 h, and between 2 h and 4 h (Figure 5c). *VEGFR2 *expression was maximal at 4 h and decreased progressively from 6 h onwards. Expression of *VE-cadherin *in D7 ESCs was maximal at 4 h, although expression was not significantly different from 0 h. Expression decreased from 6 h to 14 h and was significantly lower than 0 h between 10 h and 14 h (Figure 5d). Expression of *CD31 *in D7 ESCs was not significantly different between 0 h and 8 h (Figure 5e), decreasing significantly between 10 h and 14 h.

### The incorporation of transplanted EPCs into pre-existing endothelial tubules *in vitro *is dependent on the number of cells transplanted

**Figure 6:**
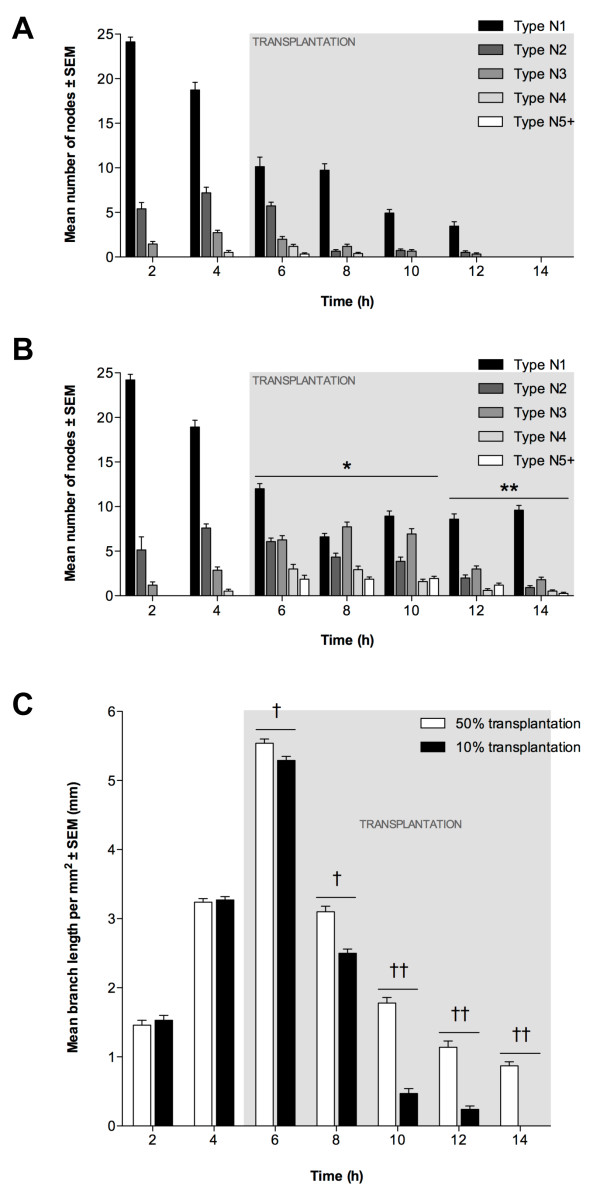
***In vitro *EPC transplantation into EC tubules**. Transplantation using 10% (8 × 10^3^) or 50% (4 × 10^4^) EPCs at 5 h. Node counts of (A) 10% and (B) 50% transplantation (± SEM, n = 3). *P < 0.05 and **P < 0.01 vs. 10% EPC transplantation at same time-point. (C): Mean tubule length per mm^2 ^(±SEM; n = 3). Significant differences between transplantations indicated (†P < 0.05, ††P < 0.01).

To determine whether EPC transplantation could enhance the longevity of existing EC tubules, we added EPCs to ECMatrix gel containing branching ECs that had been in culture for 5 h. Transplantation was performed with EPCs equal to 50% or 10% of the original number of ECs. Following transplantation with 10% equivalent EPCs, the greatest number of N1 nodes occurred at 2 h, decreasing by 6 h (Figure 6a). However, there was no significant decrease in N1 nodes between 6 h and 8 h. A progressive decrease in N1 nodes was then seen between 10 h and 12 h, and by 14 h no N1 nodes were present. The maximum number of N2 and N3 nodes occurred at 4 h (1 h prior to transplantation), whilst N4 nodes were maximal at 6 h (1 h after transplantation) and N5+ nodes were only evident at 6 h. Nevertheless, in the 50% EPC-equivalent transplantation assay, it is evident that the number of N1 nodes decreased progressively between 2 h and 8 h (Figure 6b). However, an increase in N1 nodes was seen by 10 h (P < 0.01), and between 10 h and 14 h the mean number of N1 nodes did not change significantly. The greatest number of N2 nodes was recorded at 4 h (1 h prior to transplantation) although N3 nodes were maximal at 8 h (3 h after transplantation). However, the mean number of N4 nodes, first evident at 4 h, increased significantly from 4 h to 6 h (P < 0.05), then decreased progressively between 8 h and 12 h. N5+ nodes were first identified at 6 h, with no significant difference between 6 h and 10 h with a slight reduction between 10 h and 14 h, whilst a significant decrease was observed at 14 h (P < 0.05).

Transplantation had a beneficial effect on mean branch length following both 50% and 10% transplantation. It increased from 2 h to 6 h (P < 0.01; Figure 6c) with maximum values for both assays recorded at 6 h, although this was slightly greater for the 50% transplantation (P < 0.05). For both assays, branch length decreased between 6 h and 14 h, with decreases at each time-point being significantly lower following the 10% transplantation (P < 0.01).

**Figure 7:**
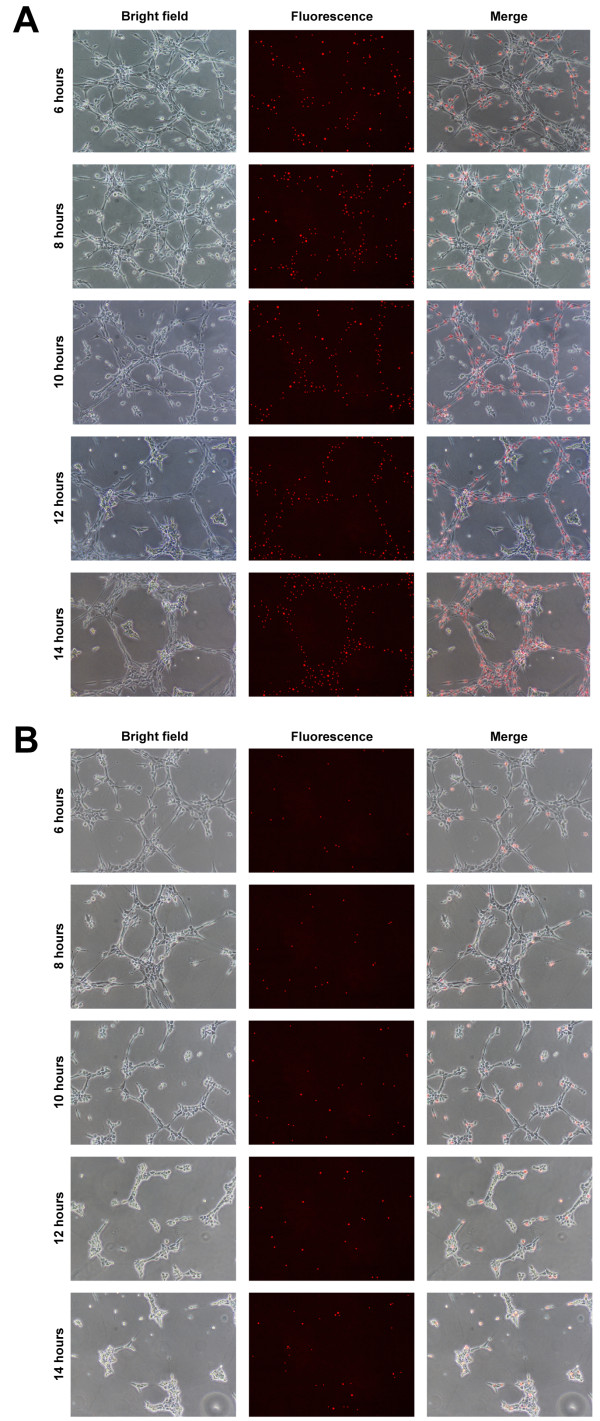
**Localisation of EPCs following in vitro transplantation**. Transplantation into EC tubules performed using 50% (4 × 104) or 10% (8 × 103) Qdot-labelled EPCs at 5 h. EPC localisation in (A) 50% and (B) 10% transplantation assays, showing bright field, fluorescence and merged images from 6-14 h at 2 h intervals. Scale bars = 500 μm.

By labeling EPCs with fluorescent Qdots prior to transplantation, their localisation into existing EC tubules could be determined. With 50% transplantation, Qdots were detected in high abundance throughout the assay at all time-points (Figure 7a). Whilst Qdots were not detected ubiquitously throughout the existing EC network, between 6 h and 14 h tubule structures were observed, composed entirely of Qdot-labelled EPCs. Following 10% transplantation, Qdots were randomly distributed within the existing EC tubule network (Figure 7b).

**Figure 8:**
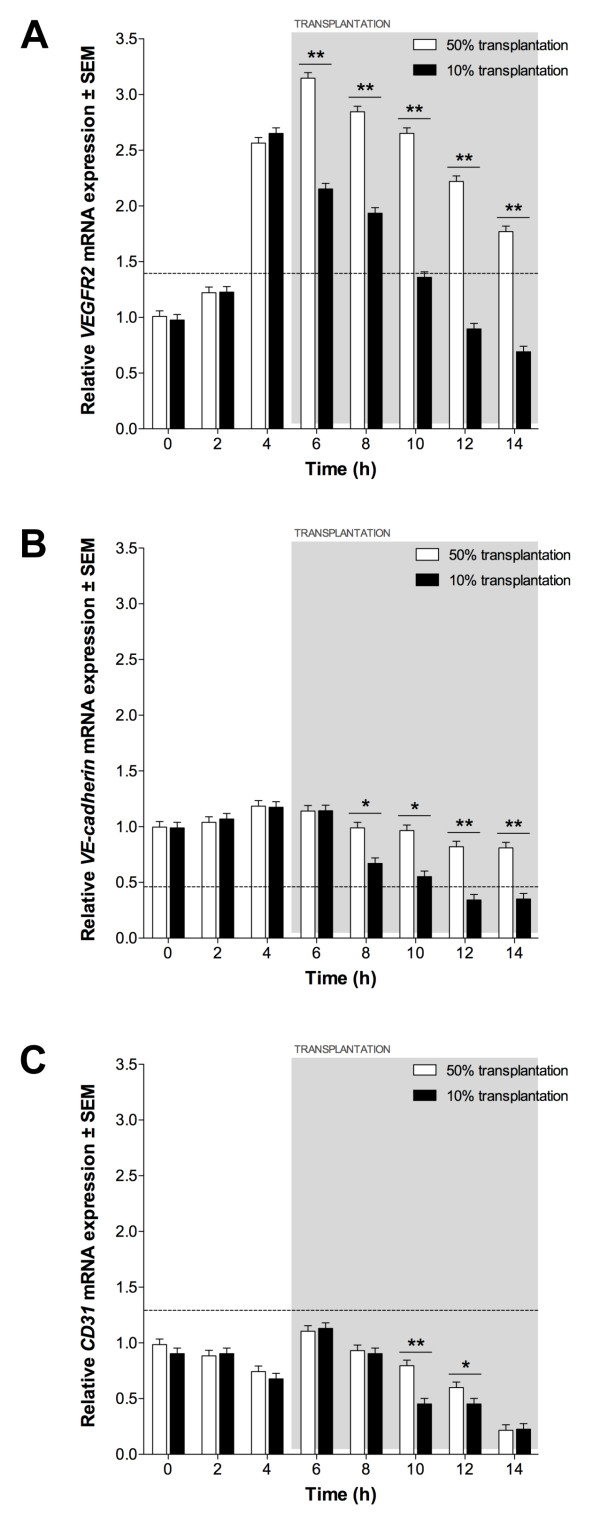
**Endothelial expression following EPC transplantation**. (A) *VEGFR2*, (B) *VE-cadherin *and (C) *CD31 *expression in 50% and 10% transplantation assays, relative to expression in 60% confluent ECs. Dotted line indicates expression in 60% confluent EPCs. Significant differences between transplantations indicated (*P < 0.05, **P < 0.01).

The effect of EPC transplantation on expression of VEGFR2, VE-cadherin and CD31 was assessed using qPCR. Prior to transplantation, expression patterns were equivalent to ECs grown on ECMatrix gels without transplantation. Following 50% transplantation, VEGFR2 expression increased significantly compared to non-transplanted ECs (P < 0.05; Figure 8a) but decreased continually following transplantation (6 h) until 14 h. VE-cadherin expression following 50% transplantation was not significantly different to non-transplanted ECs until 8 h when greater expression was detected (P < 0.05; Figure 8b). Although expression decreased from 8 h to 14 h it was still significantly greater at each time-point compared to control ECs (P < 0.05). CD31 expression was not altered significantly by 50% transplantation until 6 h, when greater expression was seen compared to control ECs (P < 0.05; Figure 8c). CD31 expression was greater than non-transplanted ECs until 14 h, at which time no significant difference was observed. 10% EPC transplantation did not result in significant changes in expression of VEGFR2, VE-cadherin or CD31 compared to non-transplanted ECs (P > 0.05).

## Discussion

Differences in endothelial-specific markers were observed between the two stages of endothelial maturation represented by the EPC and EC cell lines, with both mRNA and protein expression confirming their endothelial nature, in agreement with previous studies [8,17]. When cultured on ECMatrix gel *in vitro*, both EPCs and ECs formed complex endothelial tubule networks. It is generally assumed that tubule length and node complexity correlate with angiogenic potential [33]. Consequently, our data show that EPCs, which form tubules of greater maximum length and complexity, have a higher angiogenic potential than ECs. Having demonstrated the capacity of EPCs to form tubules structures *in vitro*, their transplantation potential was investigated.

The effect of EPC transplantation on tubule formation was variable, depending on the relative quantity of cells used. When tubules were transplanted with a quantity of EPCs equivalent to 50% of the number of ECs originally seeded onto the ECMatrix gel, the complexity of the resultant network was significantly different to non-transplanted EC-only controls. To this extent, the number of higher order nodes was greater after 5 h and branch length was increased following transplantation. As well as increasing complexity of the tubule network through the conversion of N1s to more complex structures, such transplantation also increased tubule longevity with nodes of all five types and branches being observed at 14 h, compared to control assays in which the network regressed after 12 h. In contrast, when EC tubules were transplanted with 10% EPCs no significant differences were observed in either node counts or branch lengths, suggesting little effect on either tubule longevity or network complexity.

The increase in tubule longevity seen with 50% EPC transplantation is consistent with previous investigations into the role of circulating EPCs, where they are continually released from the bone marrow to maintain the adult vasculature [34]. In addition to their role in vascular remodelling during angiogenesis, EPCs have also been shown to play an important part in the repair of defects in the EC layers of blood vessels and in the maintenance of vascular wall integrity throughout adult life [35]. The absence of a significant effect on tubule formation with 10% transplantation is interesting because, whilst EPC numbers increase during prolonged angiogenic response, their low number in peripheral blood of healthy adults may illustrate the active support of endothelial cell turnover by EPCs. Nevertheless, aging EPCs exhibit limited regenerative capacity and it may be that regular, low level release of EPCs from bone marrow is more beneficial for continued maintenance of endothelium [36]. Perhaps, if EPCs are indeed involved in subtle vascular maintenance, their effect is too modest to be demonstrated in the tubule formation assay. For this reason, one might not expect 10% EPC transplantation to have a dramatic effect on EC tubules in the assay.

As with the functional readout described above, significant alteration in endothelial-specific gene expression was only observed with 50% EPC transplantation. The effect of EPCs on tubule growth, i.e. the quantitated prolongation of complex nodal structures, is illustrated by greater numbers of higher-order nodes persisting throughout the assay period and the gene expression data. The rate of decrease of *VEGFR2, VE cadherin *and *CD31 *mRNA expression following transplantation is less than in the non-transplanted assay. This suggests a delay in downregulation of endothelial-specific gene expression observed with assay progression. No significant difference in expression was observed when EC tubules were transplanted with 10% EPCs, compared to non-transplanted ECs, further supporting the tubule quantification data.

The outcomes of the two transplantation experiments may be representative of two potential *in vivo *scenarios. First, 10% transplantation simulates the low-level, continuing replenishment of maintenance EPCs into the circulation. The phenotypic change in EPCs following 10% transplantation was minor and their effect on existing EC tubules was not significant. However, in 50% transplantation, EPC gene expression was observed to change dramatically and an obvious effect on EC tubule growth was observed, suggesting a transition from quiescent circulating EPCs to a more active, angiogenic phenotype. This active phenotype may be beneficial in a second scenario in which a large population of EPCs are mobilised, either naturally or by drug administration, from the BM in response to a greater vascular trauma, requiring a larger, more efficient EPC replenishment. Alternatively, accumulation of EPCs by cytokine targetting may require a threshold number before therapeutic effect may be realised. Indeed, the beneficial effects of EPCs have been demonstrated following the administration of cytokines, such as SDF-1 [37] and G-CSF [38], to increase the release of BM-resident EPCs into the circulation. In these studies, the massive increase in mobilised EPCs is contributing, by endothelial repopulation and chemokine production, to resultant angiogenic recovery, an effect reflected here in the 50% transplantation assay.

Our data shows that contact inhibition significantly downregulates endothelial-specific gene expression in static EPCs. EPCs intercalating into sites of vessel growth, becoming increasingly contact-inhibited, may have their capacity for neovascularisation affected in this way. If low-level circulating EPCs are subject to relatively low confluency in the bloodstream, the effect of contact inhibition may be reduced. If so, using 60% confluent EPCs (rather than 80% or 100%), as undertaken in our transplantation experiments, may better replicate the microenvironment of EPCs released into the circulation.

Using gene expression analysis, it was established that ESCs demonstrated endothelial phenotypes with different rates of differentiation. Levels of *VEGFR2 *and *CD31 *mRNA transcripts in directed-differentiated ESCs increased at a faster rate compared to spontaneously-differentiated ESCs. *VEGFR2 *expression in ECCM-treated ESCs reached a level equivalent to ECs two days earlier than in spontaneously-differentiated ESCs (D2 vs. D4), and an EPC-comparable level eight days earlier (D6 vs. D14). An EC-equivalent level of *CD31 *expression was observed two days earlier in ESCs treated with ECCM (D5 vs. D7), reaching an EPC-equivalent seven days earlier (D14 vs. D21). In contrast, the effect of ECCM on *VE-cadherin *expression was not significant: neither spontaneously- and directed-differentiated ESCs demonstrated expression similar to EPCs or ECs before D28. Unlike *VEGFR2 *and *CD31*, *VE-cadherin *expression was determined to be greater in ECs than in EPCs, and is considered indicative of later stages of endothelial maturation [39]. Thus, it is possible that more significant changes in *VE-cadherin *expression may have been seen if differentiation had been continued beyond D28, at a much later stage of endothelial development. Whilst ECCM has been demonstrated to improve endothelial differentiation rate, resulting in earlier onset of expression, the final expression pattern is comparable to naturally and spontaneously differentiated cells [40,41]. Our data supports this view, showing directed differentiation of ESCs using ECCM has the effect of significantly increasing the rate of development from a pluripotent stem cell to an endothelial-like progenitor cell, but does not affect the final phenotype (based on expression at D28).

The action of the ECCM is related to the particular combination of paracrine factors released into the medium during conditioning, such as VEGF and Ang-1 [42,43]. Furthermore, its effect can be augmented by inclusion of additional factors, such as stem cell factor and erythropoietin, modifying the efficacy of directed differentiation [40]. However, the precise combination of factors necessary for efficient directed differentiation is not yet understood and potentially important factors remain unknown [44]. Consequently, the mechanism controlling the effect of ECCM on cellular expression remains unclear. ESC-derived (D7) EPCs demonstrated *in vitro *tubule formation patterns similar to natural EPCs when cultured on ECMatrix gel. In addition, endothelial-specific gene expression in ESC-derived EPCs during tubule formation was found to be significantly similar to natural EPCs over the course of the tubule formation assay, further suggesting an equivalent endothelial phenotype and angiogenic potential.

## Conclusions

As a transplanted cell population, the angiogenic potential of EPCs is both cumulative and beneficial. We suggest that effective EPC transplantation relies on both the upregulation of the angiogenic phenotype of each individual cell and an increase in cell number overall to produce a significant beneficial effect. Small numbers of transplanted EPCs exhibit limited angiogenic activity in vitro whilst a larger population shows a significant transition in phenotype and a much greater angiogenic potential overall, demonstrating a differential response to vascular maintenance or major injury. We have also demonstrated that cells of a comparable phenotype and angiogenic potential to EPCs, which can be difficult to expand easily in vitro, can be efficiently derived by the differentiation of ESCs. Ultimately, with the proposed therapeutic benefits of EPCs, easily-maintained, ESC-derived EPCs may have potential as a rapidly-expandable and fully characterised alternative to natural EPCs for angiogenic transplantation therapy.

## List of abbreviations

Ang-1: angiopoietin-1; AF: Alexa Fluor; ANOVA: analysis of variance; bFGF: basic fibroblast growth factor; BM: bone marrow; Cyt B: cytochrome c oxidase; D: day of differentiation; DAPI: 4',6-diamidino-2-phenylindole; DIC: differential interference contrast; DMEM: Dulbecco's modified Eagle medium; DNA: deoxyribonucleic acid; EB: embryoid body; EC: endothelial cell; ECCM: endothelial cell conditioned medium; EGF: endothelial growth factor; EPC: endothelial progenitor cell; ESC: embryonic stem cell; FBS: fetal bovine serum; HLA: human leukocyte antigen; ICC: immunocytochemistry; LIF: leukemia inhibitory factor; MEF: murine embryonic fibroblast; N: node type; NA: numerical aperture; Qdot: quantum dot; qPCR: quantitative real-time polymerase chain reaction; RNA: ribonucleic acid; VEGF: vascular endothelial growth factor; VEGFR2: vascular endothelial growth factor receptor 2; vWF: von Willebrand Factor.

## Competing interests

The authors declare that they have no competing interests.

## Authors' contributions

PR performed the experiments, analysed the data, and wrote and approved the final manuscript; RK performed the experiments, analysed the data and approved the final manuscript; SE conceived the experiments, analysed the data, wrote and approved the final manuscript and obtained funding for the work; JS conceived the experiments, analysed the data, wrote and approved the final manuscript and obtained funding for the work.
